# Mice Overexpressing β-1,4-Galactosyltransferase I Are Resistant to TNF-Induced Inflammation and DSS-Induced Colitis

**DOI:** 10.1371/journal.pone.0079883

**Published:** 2013-12-05

**Authors:** Valerie Vanhooren, Roosmarijn E. Vandenbroucke, Sylviane Dewaele, Evelien Van Hamme, Jody J. Haigh, Tino Hochepied, Claude Libert

**Affiliations:** 1 Department for Molecular Biomedical Research, VIB, Ghent, Belgium; 2 Department of Biomedical Molecular Biology, Ghent University, Ghent, Belgium; INSERM, France

## Abstract

Glycosylation is an essential post-translational modification, which determines the function of proteins and important processes such as inflammation. β-1,4-galactosyltransferase I (βGalT1) is a key enzyme involved in the addition of galactose moieties to glycoproteins. Intestinal mucins are glycoproteins that protect the gut barrier against invading pathogens and determine the composition of the intestinal microbiota. Proper glycosylation of mucus is important in this regard. By using ubiquitously expressing βGalT1 transgenic mice, we found that this enzyme led to strong galactosylation of mucus proteins, isolated from the gut of mice. This galactosylation was associated with a drastic change in composition of gut microbiota, as TG mice had a significantly higher Firmicutes to Bacteroidetes ratio. TG mice were strongly protected against TNF-induced systemic inflammation and lethality. Moreover, βGalT1 transgenic mice were protected in a model of DSS-induced colitis, at the level of clinical score, loss of body weight, colon length and gut permeability. These studies put βGalT1 forward as an essential protective player in exacerbated intestinal inflammation. Optimal galactosylation of N-glycans of mucus proteins, determining the bacterial composition of the gut, is a likely mechanism of this function.

## Introduction

Glycosylation has been known to be important not only for correct development of the intestine but also for its proper functioning and especially for maintenance of the protective function of the mucus layer [1]. From stomach to rectum, the entire mucosa consists of a single layer of columnar epithelial cells, covered by a layer of secreted mucus which is mainly produced by specialized secretory cells known as goblet cells. The mucus layer is a protective barrier which protects the epithelial cells and underlying host tissues against direct contact with commensal microbiota of the gut. Mucus is a complex solution rich in secreted mucins [2]. These are large, heavily glycosylated glycoproteins that assemble into homo-oligomers that give mucus its viscous properties [3]. The apical surface of mucosal epithelial cells is covered by a complex glycocalyx containing large amounts of cell surface transmembrane mucins. The extracellular heavily glycosylated part of mucins can be shed into the mucus [4].

β-1,4-galactosyltransferase I (βGalT1) is an enzyme that plays a major role in glycosylation. It is transcribed, in mice, by the *B4galt1* gene and it conjugates activated galactose in a β-1,4 bond to the outer arm of N-acetylglucosamine in type 2 N-glycans [5]. B-1,4 galactosylation of glycoproteins occurs in many mammalian tissues and is involved in various physiological functions through interactions with selectins, galectins, asiologlycoprotein receptor and other molecules [6].

Growth of βGalT1-knockout mice is retarded and about 50% of the mice die before weaning, due to augmented proliferation and abnormal differentiation of the intestinal epithelial cells [7]. Additionally, βGalT1 knockout mice display peripheral blood leukocytosis and reduced acute and chronic inflammatory responses [8], which shows that βGalT1 plays an important role in inflammation. Links between tumor necrosis factor (TNF), a very potent proinflammatory molecule and a key signaling component of the immune system [9] and βGalT1 expression are known. First, stimulation of several cell types with TNF *in vitro* increases βGalT1 expression [10]–[12]. Second, i*n vivo*, spinal cord contusion injury as well as injection of LPS into the spinal cord induce βGalT1 expression, and it was suggesting that TNF might play a role in regulating βGalT1 expression. However, direct induction of βGalT1 gene expression by TNF *in vivo* has not been demonstrated [13], [14].

Inflammatory bowel disease (IBD) is a TNF-dependent, immune-mediated disease resulting from a disturbed interaction between gut microbiota and mucosal cells in genetically susceptible hosts [15], [16]. The role of TNF in IBD is well established and TNF is a successful drug target in IBD. Also, TNF is a mediator in sepsis and we recently proved that the gut is an essential target in TNF's induction of systemic inflammatory response syndrome (SIRS), in a sepsis-like mouse model [17]. Deterioration of the mucus layer of the colon is commonly observed in patients with ulcerative colitis, a common form of IBD [18], [19]. The importance of mucus in protecting against IBD is supported by different mouse models, e.g. Winnie mice, which have a missense mutation in the *Muc2* mucin gene, and Muc2^−/−^ mice, which develop spontaneous colitis [20], [21]. Besides N-glycans, also O-glycans in mucus are known to be essential for protection against colitis. Mice lacking core-3- and core-2-derived O-glycans are more susceptible to colitis [22], [23]. Mice deficient in core-1-derived O-glycans even spontaneously develop colitis [1]. But hardly any studies have been performed on the relevance and importance of N-glycosylation, and particularly galactosylation, during colitis [24].

To investigate the role of βGalT1 in TNF-mediated diseases such as sepsis and IBD, we generated mice overexpressing βGalT1 (TG) and subjected them to TNF-induced SIRS and DSS-induced colitis. We demonstrate that βGalT1 TG mice are resistant to TNF-induced lethal SIRS and that βGalT1 expression in the gut of WT mice increases after TNF injection. Likewise, TG mice are more resistant to DSS-induced colitis. We hypothesize that the main mechanism for these effects is the protective effect of fully galactosylated mucus in the intestine of the TG mice, which leads to a healthier Firmicutes/Bacteroidetes ratio.

## Results

### Generation and phenotyping of mice overexpressing βGalT1 (TG)

**Figure 1:**
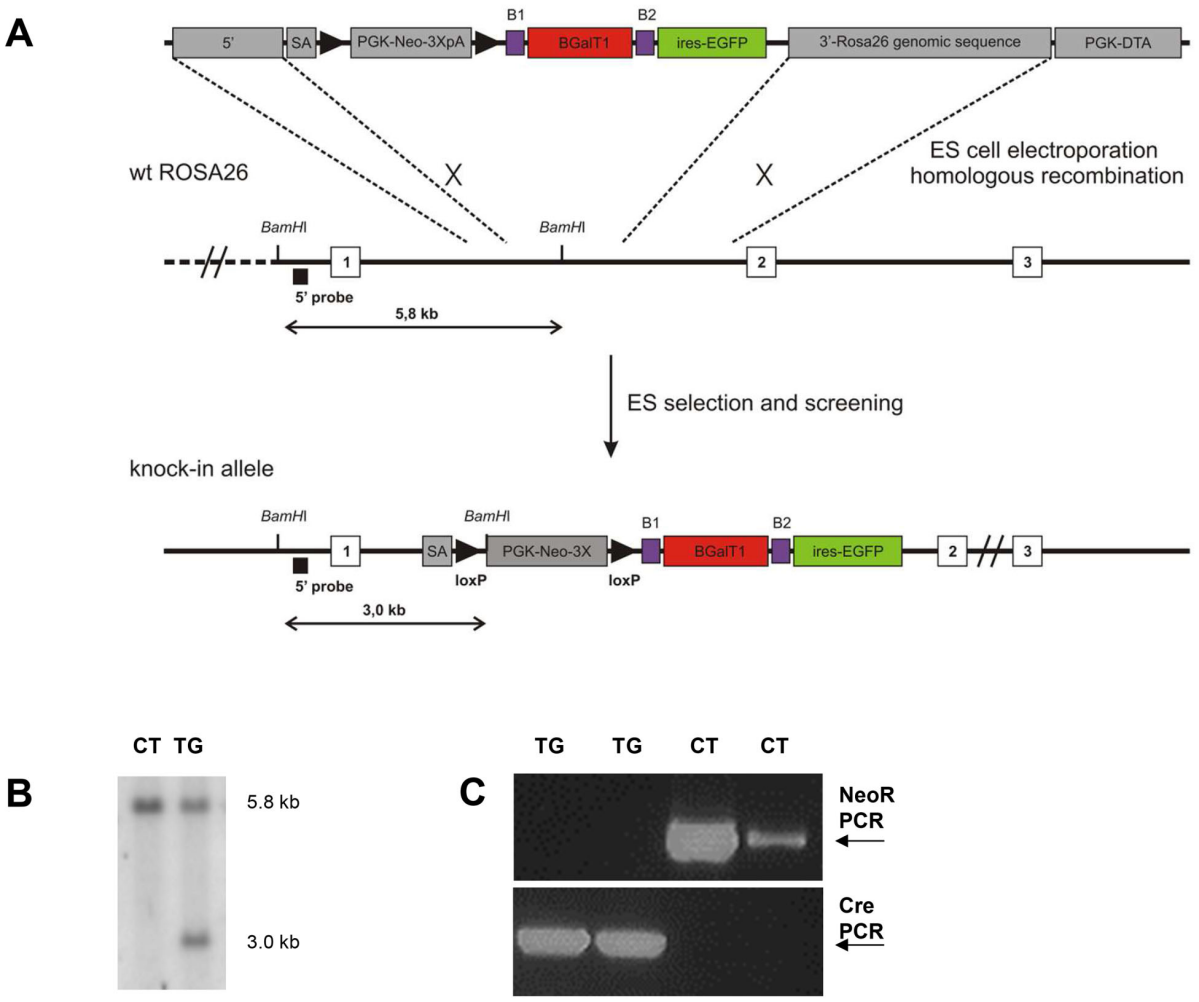
Generation of TG mice overexpressing βGalT1. (A) ES-cell targeting strategy, adapted from [25]. Restriction enzyme sites and the location of the probe used for Southern blot analysis are depicted. The ROSA26 locus was targeted using the targeting vector (top) by homologous recombination. After antibiotics selection and Southern blot analysis, the ROSA26 locus was modified as shown in the figure. (B) Southern blot analysis on DNA derived from WT and heterozygous recombinant ES cells after *BamH*1 digestion and using the probe depicted in panel A. (C) PCR analysis on tail-extracted genomic DNA of two TG mice (with floxed βGalT1 alleles and containing cre) and two CT mice (mice with floxed βGalT alleles but not containing cre). PCR on genomic DNA was performed to detect the floxed Neomycin resistance gene (upper panel) and the cre gene (lower panel).

Using the Gateway-compatible ROSA26 locus targeting vector [25], we generated conditional βGalT1-overexpressing mice by knocking in the mouse βGalT1 cDNA preceded by a loxP-flanked stop cassette under control of the ROSA26 promoter (Fig. 1A). Correctly targeted embryonic stem cell clones were identified by Southern blotting and were used to generate chimeric mice that transmitted the transgene to their offspring (Fig. 1B). Mice homozygous for the loxP-flanked stop cassette were used as control mice (CT). They did not display altered βGalT1 expression compared to WT mice (data not shown). CT mice were crossed with deleter-cre mice expressing Cre in all cell types [26]. Mice heterozygous for the transgene and containing one Cre copy were intercrossed to obtain the homozygous transgenic mice (TG) which were used in all experiments. Efficiency of excision of the stop cassette by Cre was confirmed by performing PCR on tail genomic DNA for Cre and for the stop cassette (Fig. 1C).

The TG mice were born without obvious defects and were found to have 2 fold higher expression and activity levels of βGalT1 in all the organs tested (Fig. S1).

**Figure 2:**
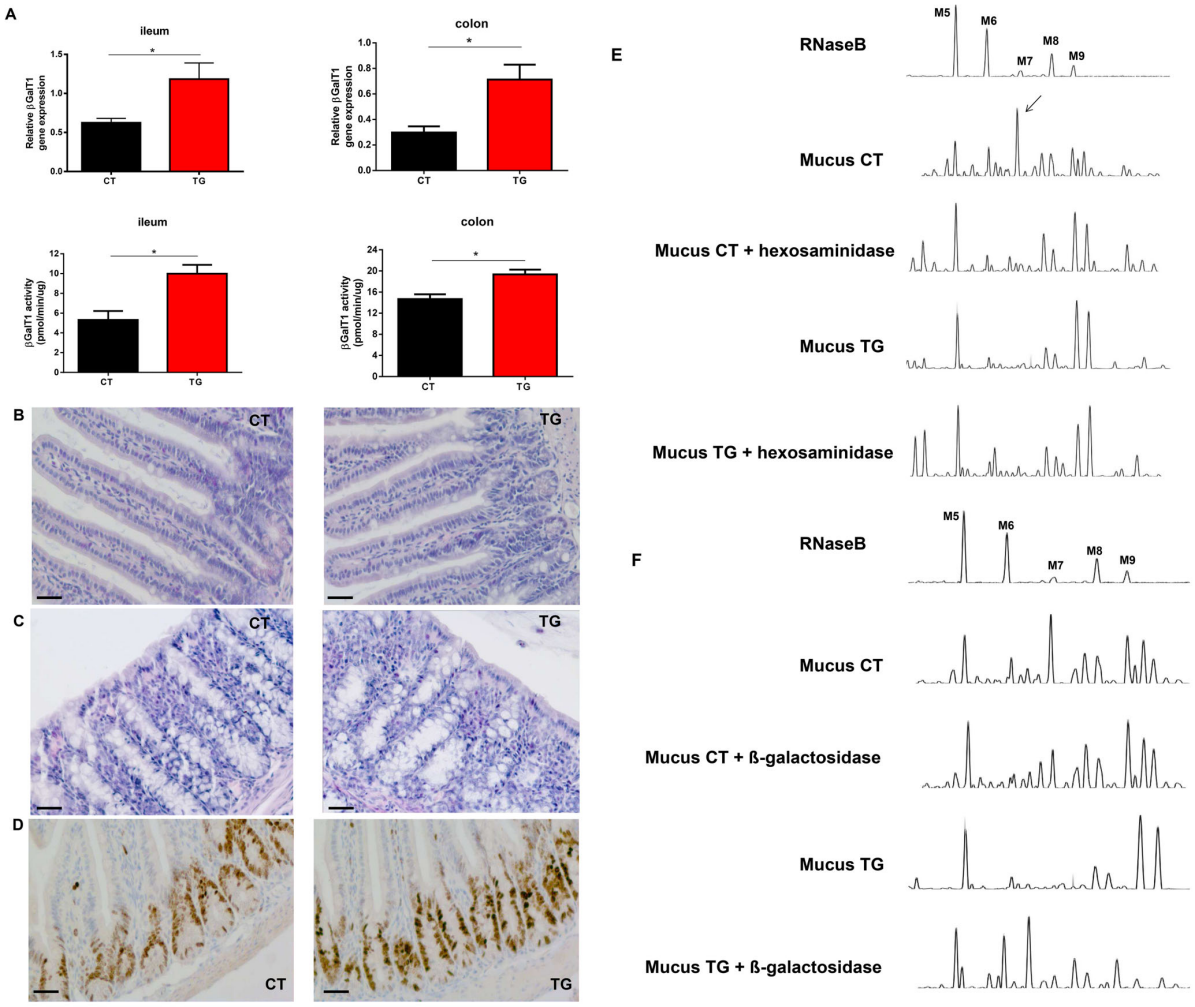
Basic characteristics of TG mice overexpressing βGalT1. (A) Relative βGalT1 gene expression and βGalT1 activity in the ileum and colon mucosa of βGalT1-overexpressing mice (TG) mice (black, n = 5) is 2 fold higher compared to CT mice (red, n = 6). Top left βGalT1 QPCR in ileum mucosa and top right βGalT1 QPCR in colon mucosa. Below left βGalT1 activity in ileum and below right βGalT1 activity in colon. Representative H&E-stained (B) ileum and (C) colon mucosa from CT (left) and TG (right) mice. The bar in the left corner represents 50 µm. No morphologic alterations are observed in the TG mice. (D) Ki67-stained ileum mucosa from CT (left) and TG (right) mice. The bar in the left corner represents 50 µm. No changes in proliferation rate are observed in the TG mice. (E) Analysis of the N-glycosylation profile of ileum mucus from mice with and without hexosaminidase treatment. Top panel is the N-glycosylation profile of a standard protein, RNase B. The second panel represents a representative sample of ileum mucus of a CT mouse with a high peak running at the same position as M7 of RNaseB. The third panel represents the N-glycosylation profile of CT mucus (the same sample as shown in panel 2), but after hexosaminidase treatment, which demonstrates that the peak running at M7 position contains terminal GlcNAc. The fourth panel is a representative sample of ileum mucus of a TG mouse, lacking the M7 peak. The fifth panel is the same sample as in the fourth panel but after hexosaminidase treatment, demonstrating that TG mucus has less structures ending with GlcNac compared to CT mucus. (F) Analysis of the N-glycosylation profile of ileum mucus from mice with and without ß-galactosidase treatment. Top panel is the N-glycosylation profile of RNase B. The second panel represents a typical sample of ileum mucus of a CT mouse. The third panel is the same sample as the second panel but treated with ß-galactosidase. After isolation of the N-glycans out of mucus, these N-glycans are treated with ß-galactosidase in order to identify terminal galoctose residues in the sample since ß-galactosidase only cleaves terminal galactose. Only a few peaks shift, which demonstrates that only a small amount of the N-glycans in CT mucus have terminal galactose. The fourth panel is a representative sample of ileum mucus of a TG mouse. The fifth panel is the same sample as in the fourth panel but treated with ß-galactosidase. Here a clear shift of peaks is observed demonstrating that the N-glycans in TG mucus have more terminal galactose compared to the CT mucus.

Next, we focused on the gut because proliferation of the intestinal epithelial cells in βGalT1^−/−^ mice is augmented and their differentiation is abnormal [7]. Mucosa of the small intestine and colon were isolated from CT and TG mice and mRNA was prepared. TG mice expressed a two-fold higher level of βGalT1 mRNA than CT mice in the mucosa of ileum and colon (Fig. 2A). βGalT1 activity assays demonstrate a similar trend as the βGalT1 gene expression namely a two-fold higher βGalT1 activity is present in the ileum and colon of TG compared to CT mice (Fig. 2A). As judged by H & E staining, this increased expression and activity did not alter the mucosa morphology in ileum and colon (Fig. 2B–C) nor the proliferation rate in the mucosa of ileum, as assessed by Ki67 staining (Fig. 2D).

It has been reported that βGalT1^−/−^ mice display basal peripheral blood leukocytosis [8], so blood composition was analyzed, but no differences between TG and CT mice could be detected (Fig. S2). These data show that the TG mice display no obvious abnormalities in the composition of the mucosa of the gut.

Proper functioning of intestinal mucus depends on its glycosylation. It has been reported that N-glycosylation of mucins is important for folding, oligomerization and surface localization of these proteins [27]. Recent studies showed that O-glycosylation of mucus is essential for its functionality, but little is known about functional importance of N-glycosylation of mucins [1], [22]. To address this, we visualized the goblet cells, the major mucus-producing cells of the intestine, by Alcian blue staining in the small intestines of CT and TG mice. However, no difference in amount, size or localization of goblet cells could be observed (Fig. S3A). We also measured the total amount of mucus present in ileum of CT and TG mice. Also here, no difference in amount of mucus per cm ileum could be detected (Fig. S3B). Next, we performed a qualitative analysis of the mucus. We isolated the mucus from the ileum, isolated all N-glycans, and separated them according to size by the DSA-FACE, a technique widely validated and used to study N-glycosylation [28]–[33]. A clear difference in ileum mucus N-glycosylation profile was observed between CT and TG mice (Fig. 2E). The mucus of CT mice displayed a very high peak, running with the same mobility as M7 of the reference RNaseB, which is the mobility position of non-galactosylated structures in human serum, while in the mucus of TG mice, this peak was absent. To confirm that this peak represents N-glycans that are not terminally galactosylated and possibly are GlcNAc-terminating acceptors, we performed a glycodigest with hexosaminidase, an enzyme that cleaves GlcNAc only if it is attached terminally. This enzymatic cleavage reduced the peak running around M7, which confirms that this peak contains a structure that does not end with galactose but with GlcNAc (Fig. 2E). Also other peaks present in CT mucus have a terminal GlcNAc since more peaks (not only the one running at same mobility as M7) shift after hexosaminidase treatment. In contrast, hexosaminidase digestion of TG N-glycans does also alter the profile, but to a far lesser extent demonstrating that a 2 fold βGalT overexpression increases the galactose terminating structures in mucus but is not sufficient to cover all terminal GlcNAcs (Fig. 2E). To confirm that more N-glycans of TG mice are terminally galactosylated compared to CT mice, β-galactosidase digests were performed on the same sugars. β-galactosidase is an enzyme that cleaves galactose only if it is attached terminally. Treating CT sugars with β-galactosidase modified the CT profile to some extent but severely less than the TG sugars treated with β-galactosidase, where a total shift of peaks occurred (Fig. 2F). This demonstrates again that a 2 fold overexpression of βGalT increases the galactose terminating structures in the mucus of ileum from the TG mice. We observed a similar increase in galactose terminating N-glycans in colon mucus of TG mice compared to CT mice (Fig. S3C–D).

### βGalT1 modulates the gut microbiota composition

Glycans on mucins are necessary for interaction between mucus and microbial lectins. Hence, altered mucin glycosylation can modify microbiota composition [34]. The gut microbiota is a crucial protective component of the gut mucosa, and it is also thought to contribute to gut tissue repair [35].

**Figure 3:**
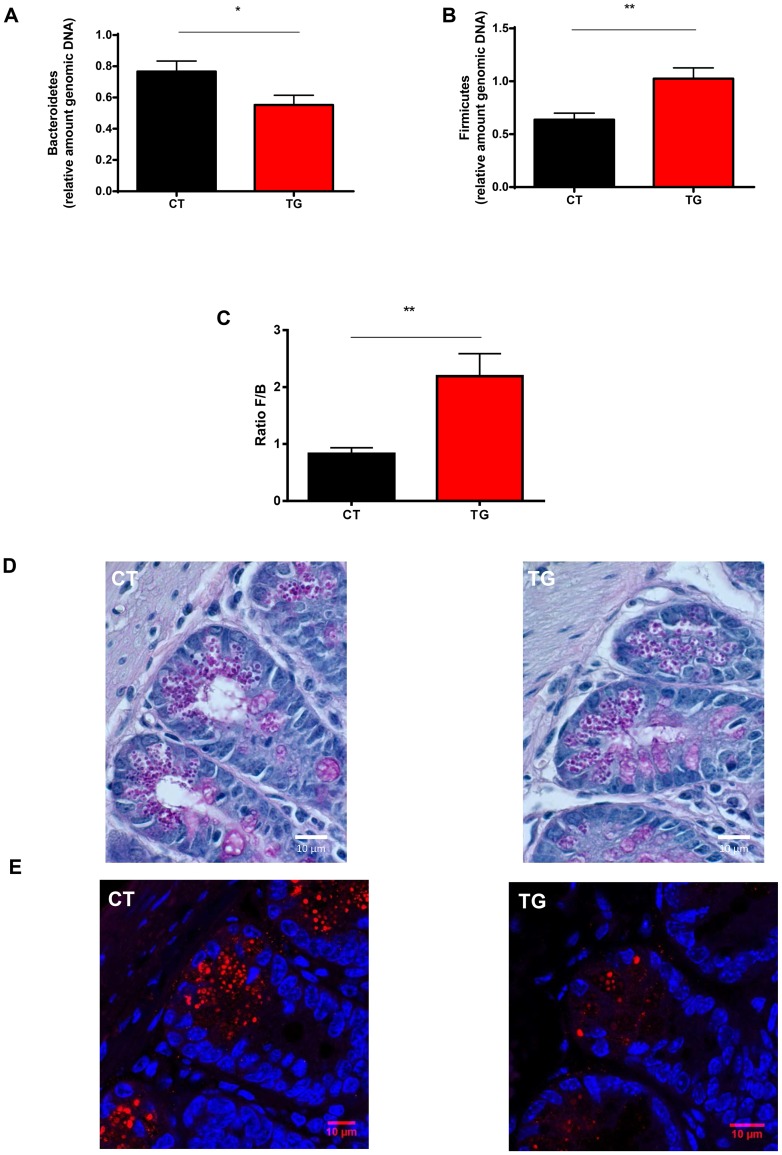
Microbiota composition in CT and βGalT1 mice. Fecal bacterial DNA was isolated from CT mice (black, n = 12) and TG mice (red, n = 12) and qPCR was performed with primers specific for the main bacterial species normally found in the gut. After the qPCR, the results were normalized to the total amount of bacteria. (A) Bacteroidetes are reduced and (B) Firmicutes are increased in the TG mice compared to CT mice. (C) The Firmicutes to Bacteroidetes ratio is significantly increased in TG mice compared to CT mice. (D) Visualization of the Paneth cell numbers in ileum by PAS staining. Representative CT sample on the left and TG sample on the right. TG mice have similar amounts of Paneth cells compared to CT mice. (E) Visualization of the Paneth cells granules by MMP-7 staining in ileum. Representative CT sample on the left and TG sample on the right. TG Paneth cells have reduces granule content compared to CT Paneth cells.

As we demonstrated that the N-glycan profile is significantly altered in mucus of TG mice, we compared the microbiota composition of TG mice and CT mice. Bacterial DNA was isolated from mouse feces and qPCR was performed with primers specific for the main bacterial species groups found in the gut, and the results were normalized to total amounts of bacteria. The total amount of bacteria was similar in CT versus TG mice (data not shown). We did found fewer Bacteroidetes and more Firmicutes in TG mice than in CT mice (Fig. 3A–B). TG mice had a significantly higher Firmicutes to Bacteroidetes ratio (Fig. 3C). We demonstrate that increased βGalT1 expression can significantly alter this ratio to a more protective microbial constitution, since reduced counts of Firmicutes are known to be consistently associated with reduced protection of the gut mucosa, as seen in IBD patients, infants and elderly individuals [35], [36].

Furthermore, we analyzed if this altered Firmicutes to Bacteroidetes ratio had an impact on Paneth cell composition. Paneth cells in mouse small intestinal crypts secrete granules rich in microbicidal peptides when exposed to bacteria or bacterial antigens [37]. We visualized the Paneth cells by PAS and MMP-7 staining [38], [39]. The amount of Paneth cells are similar in CT and TG ilea (Fig. 3D). TG Paneth cells do have reduced granule content compared to CT Paneth cells (Fig. 3E) suggesting there is a reduced need of anti-bacterial peptide production in the TG mice due to a “healthier microbiota” composition.

Next, we analyzed whether this altered microbiota composition is protective in inflammation models by subjecting CT and TG mice to TNF-induced SIRS, a model in which the gut is a crucial target [17] and DSS-induced colitis.

### βGalT1-overexpressing mice are resistant to TNF-induced inflammatory shock

**Figure 4:**
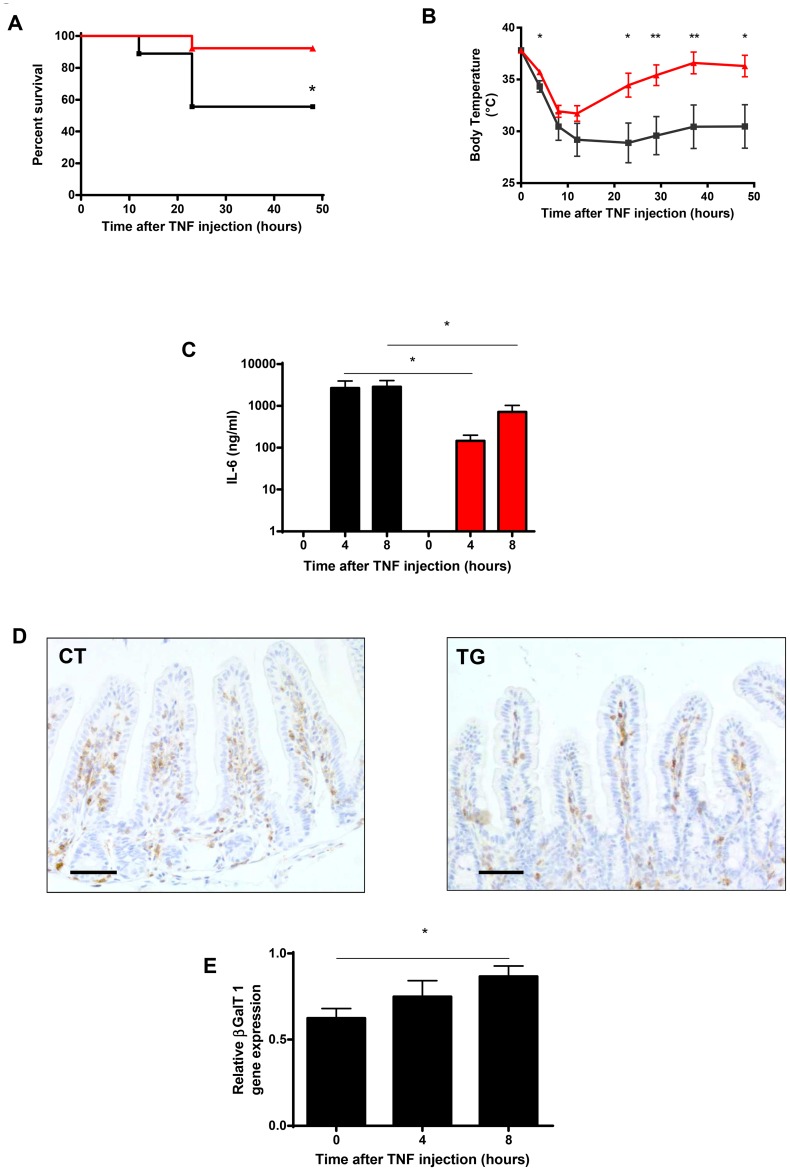
Mice overexpressing βGalT1 are protected against TNF-induced inflammatory shock. TNF (20 µg/mouse) was injected in CT (black line, n = 9) and TG (red line, n = 13) mice. Survival (A) and body temperature (B) were followed during the experiment over a period of 50 h. No further deaths occurred. (C) IL-6 serum levels were measured in CT mice (black bars, n = 5) and TG mice (red bars, n = 7), 4 hours and 8 hours after TNF injection. Staining of CD45 positive white blood cells in the ileum of CT (D left) and TG mice (D right) 4 hours after TNF challenge, by immunohistochemistry. A representative CT and TG sample are shown. The bar in the left corner represents 50 µm. No significant difference in the number of CD45 positive cells per surface area was observed. (E) Relative βGalT1 gene expression, measured by qPCR in mucosal samples from CT mice after injection with recombinant mouse TNF (20 µg/mouse), is upregulated in function of time.

TNF causes severe damage to the intestine [40], and the intestine is an important regulator of TNF-induced shock [17], [41], [42]. Also, depleting bacteria by antibiotic treatment reduces the severity of TNF-induced inflammation and intestinal damage [43]. We investigated whether increased mucus galactosylation and consequent increased Firmicutes/Bacteroidetes ratio's alters TNF toxicity. We injected CT and TG mice with TNF and monitored survival (Fig. 4A) and body temperature (Fig. 4B). Inflammation was monitored by serum IL-6 levels (Fig. 4C) [44].

In terms of survival, a significant protection against TNF was found in the TG mice: most TG mice survived the challenge, while half of the CT mice died (Fig. 4A). This different response was also reflected in body temperature. While both groups displayed an initial drop in body temperature, TG mice recovered much faster than CT mice. Furthermore, IL-6 serum levels after TNF injection were significantly lower in TG mice than in CT mice. This demonstrates that TG mice experience less severe systemic inflammation and cope better with TNF-induced inflammatory shock. Because βGalT1^−/−^ mice were shown to suffer from peripheral blood leukocytosis and reduced inflammatory responses caused by impaired rolling of white blood cells [8], we analyzed the peripheral white blood cell composition in CT and TG mice after TNF injection, but no differences were found (data not shown). Similarly, we detected the same amount of CD45 positive white blood cells per surface area in the ileum of CT and TG after TNF challenge (for representative examples see Fig. 4D). Our results suggest that overexpression of βGalT1 reduces TNF-induced inflammation at the level of the intestines but does not directly influence the infiltration capacity of leukocytes.

### TNF induces βGalT1 expression in vivo in the mucosa of the gut

TNF-induced βGalT1 expression has been demonstrated *in vitro* in astrocytes, Schwann cells and fibroblast-like synoviocytes [10]–[12]. To study the effect of TNF on βGalT1 expression *in vivo*, we injected CT mice with TNF, isolated ileum mucosa and examined βGalT1 gene expression by qPCR. We focused on the ileum mucosa because the small intestine is a main target of TNF in the process of TNF-induced shock [40], [43], [45], [46]. We found that βGalT1 expression is induced in the mucosa as early as 4 h after TNF injection and that its expression is even higher 8 h post-injection (Fig. 4E). Although this increase is limited it is clearly significant indicating that TNF is an inducer of βGalT1 gene expression *in vivo*.

### βGalT1 TG mice display reduced susceptibility to DSS-induced colitis

The fecal microbiota composition of patients with IBD differs from that of healthy individuals. qPCR and sequencing analysis shows that Firmicutes bacteria in particular are underrepresented in IBD patients [35]. Firmicutes bacteria have anti-inflammatory properties: oral administration of live Firmicutes bacteria reduced the severity of TNBS colitis in mice [47]. Therefore, a high ratio of Firmicutes to Bacteroidetes is thought to be protective against colitis.

**Figure 5:**
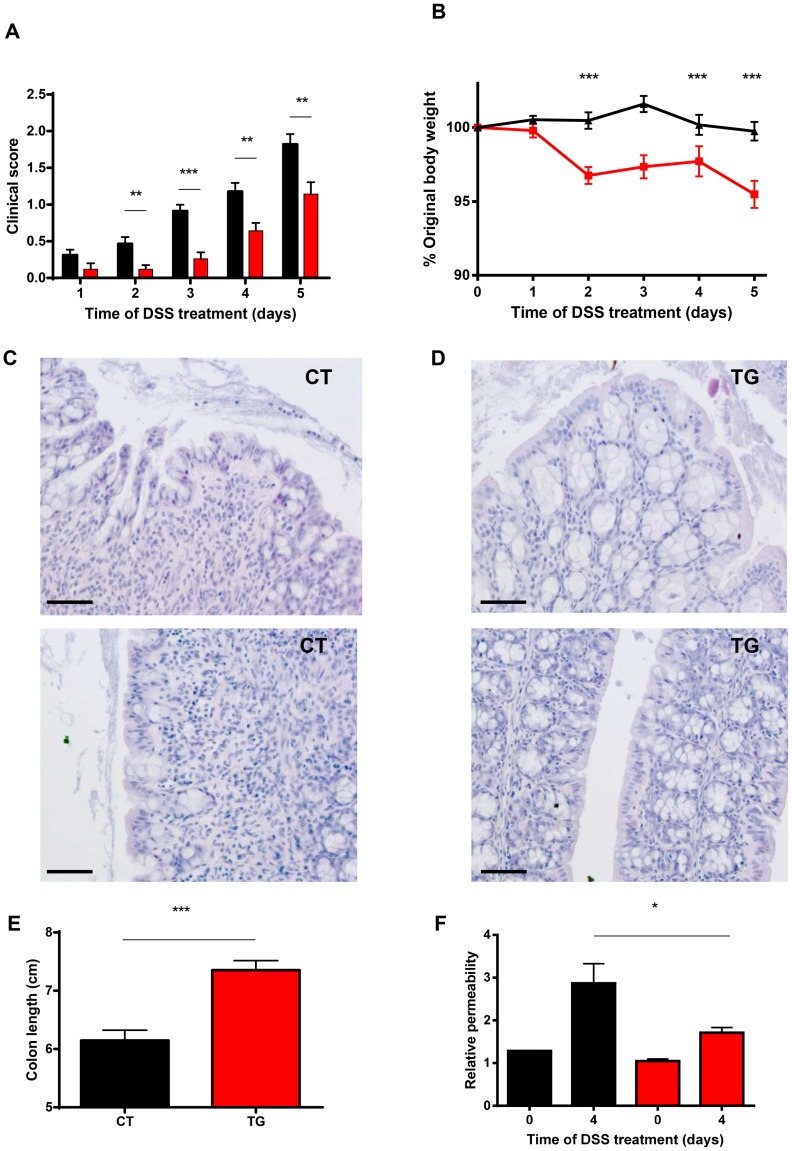
Mice overexpressing βGalT1 are protected against DSS-induced colitis. (A) Clinical score and (B) body weight of CT (black, n = 20) and TG (red, n = 20) mice treated with 3% DSS in the drinking water. H&E staining of the colon of (C) CT mice and (D) TG mice after 4 days of DSS treatment. Two representative CT and TG sample are shown. The bar in the left corner represents 50 µm. (E) Colon length of CT mice (black, n = 15) and TG (red, n = 15) mice after 4 days of 3% DSS treatment. (F) Determination of DSS-induced gut permeability in CT (black, n = 17) and TG (red, n = 14) mice. FITC-dextran was administered orally to both groups of mice, 4 days after the start of DSS treatment. Five hours after gavage, blood was taken and fluorescence in plasma was measured. Two control CT and TG mice, not treated with DSS, but only with FITC-dextran were included. Fluorescence in plasma increased due to leakage of FITC-dextran from the intestine into the blood significantly more in CT than TG mice.

As we detected a higher ratio of Firmicutes to Bacteroidetes in gut microbiota of TG mice and these mice were found to be significantly less susceptible to TNF, the toxicity of which clearly involves the gut [17], we compared CT and TG mice in a TNF-mediated gut inflammatory model, i.e.colitis induced by dextran sulfate sodium (DSS) [15]. Many morphological and pathophysiological features of DSS-induced colitis are similar to those observed in human ulcerative colitis, such as the production of inflammatory cytokines and loss of barrier function [48], [49]. Mice received 3% DSS in the drinking water for 5 days, and body weight, diarrhea and rectal bleeding were monitored daily, yielding a clinical score. Compared to CT mice, TG mice developed less severe symptoms of colitis, such as milder gross rectal bleeding and diarrhea, leading to a lower clinical score (Fig. 5A). Also, the TG mice were protected against loss of body weight (Fig. 5B). In agreement with the clinical observations, there were clear morphological differences in the colons of CT versus TG mice after 4 days of DSS treatment. The CT colons demonstrate a severe loss of crypt architecture and overall structure of the lamina propria and a significant decrease in goblet cell amounts (Fig. 5C); this was not seen in the colons of TG mice 4 days after DSS treatment (Fig. 5D).

Additionally, colon shortening, a typical clinical feature of colon inflammation and of the DSS model, was significantly more pronounced in CT mice than in TG mice, after 4 days of DSS treatment (Fig. 5E). Colon permeability has been shown to be a critical step in susceptibility to colitis [50]. Therefore, we measured gut leakage in CT and TG mice after 4 days of DSS treatment. FITC-dextran was administered orally to both groups of mice and five hours after gavage, blood was taken and fluorescence was measured in the plasma, as described before [51]. We observed no differences in basal gut permeability, but a significantly reduced increase in gut permeability in the TG mice compared to the CT mice (Fig. 5F). Taken together, these results demonstrate that βGalT1 TG mice are strongly protected against DSS-induced gut barrier disruption and other DSS-induced phenomena. In conclusion, βGalT1 overexpression protects against TNF-mediated inflammatory colitis.

## Discussion

Oligosaccharides of glycoproteins play important biological roles by influencing the functions of the proteins to which they are attached [52]. They are important for maintaining the complex life of cells in multicellular organisms by modulating cell adhesion, self/nonself recognition, molecular trafficking and many other functions [53], [54].

βGalT1, is very important in the glycosylation process since, in the Golgi apparatus, it transfers galactose from uridine diphosphate galactose to terminal GlcNAc of carbohydrate chains in a β-1,4-linkage to form a Galβ1-4GlcNAc structure [55]. βGalT1 is expressed ubiquitously, suggesting that it is involved in β4 galactosylation of many glycoproteins. β4 galactosylation of glycoproteins is widely distributed in mammalian tissues and is involved in various physiological functions [14], [56], [57].

To find the true biological function of βGalT1 in mammals, mutant mice can be generated. βGalT1-deficient mice have been described. We here describe, for the first time, βGalT1 overexpressing mice. In βGalT1-deficient mice, inflammatory responses are suppressed and neutrophil infiltration at inflammatory sites is reduced [8] but also skin wound healing is significantly delayed [58]. Although βGalT1^−/−^ mice are useful for studying the function of βGalT1, they also demonstrate developmental defects, such as growth retardation, and the proliferation and abnormal differentiation of the intestinal epithelial cells [7]. To circumvent these developmental abnormalities and to generate a novel and interesting complementary tool, we generated βGalT1-overexpressing mice and studied their response in inflammatory models involving the gut. The mice were born normally without obvious defects, and their blood leukocyte compositions and counts were normal. In contrast to βGalT1^−/−^ mice, our TG mice showed no abnormal differentiation or proliferation of the mucosa but a difference in the N-glycosylation composition of the mucus was observed. While CT mucus contains glycoproteins with N-glycans terminating with GlcNAc (so without terminal galactose), TG mucus glycoproteins are almost fully galactosylated. This demonstrates that under normal physiological conditions βGalT1 cannot perform galactosylation of all mucus N-glycoproteins. By overexpressing βGalT1 some 2–3 fold, almost all N-glycoproteins in the mucus become N-galactosylated.

Mucus is a complex, gel-like fluid that forms a continuous protective layer over the surface of the gastrointestinal mucosa from stomach to rectum. It protects the delicate underlying intestinal epithelial cells (IEC) against pathogens from the lumen [59]. Mucus is rich in mucin glycoproteins that are heavily N- and O- glycosylated [2]. On the other hand, the mucus layer provides an energy source for the endogenous microbiota that colonize it, and thereby the mucus sustains the health status of the gut microbiota, [60] which is important since the gut microbiota contribute to host nutrition and energy balance and to the development and maintenance of a robust immune system [61].

Different bacterial species groups bind to different N-glycan epitopes [62], [63]. Therefore, a change in glycosylation of the mucosa can influence the binding of bacteria to it. For example, mice lacking the enzyme fucosyltransferase-2 display altered glycosylation of mucins leading to altered binding of *Helicobacter pylori* to the gastric mucosa [64]. We therefore studied the effect of increased mucus galactosylation in TG mice on the gross microbiota composition. In TG mice, Firmicutes were increased and Bacteroidetes were decreased, which resulted in a very significantly higher Firmicutes/Bacteroidetes ratio. Such high ratios have been postulated to be protective against IBD [35]. Thus changes in the expression of a single glycosyltransferase can have a large effect on the crucial composition of the gut microbiota. Inversely, it has been demonstrated that bacteria can induce alterations in the expression of host glycosyltransferases and glycosidases in the gut [65]. Further study is required to determine whether Firmicutes can induce the expression of βGalT1 or if other commensal bacteria can specifically regulate the galactosylation state of the mucus. Such studies can shed light on how the composition of microbiota is determined and how it can be regulated and manipulated.

The TG mice have less anti-microbial products in their Paneth cells. This could be caused by a reduced need of anti-bacterial protein production in the TG mice due to a “healthier microbiota” composition. Few studies discuss how microbiota composition influences Paneth cell anti-bacterial peptide production [66]. How microbial composition influences Paneth cell granule composition in the intestine should be studied further in depth.

To study if this altered N-glycosylation profiles and the microbiota compositions of the gut are protective against TNF-mediated inflammatory responses, we tested TG mice in inflammatory models in which the gut and the microbiota play a crucial role [15], [17], [42]. Commensal bacteria are crucial for TNF-induced toxicity. Depletion of bacteria by antibiotics reduces the severity of TNF-induced gut inflammation and damage [17], [43]. Our TG mice survived an otherwise lethal injection of TNF and developed milder inflammation than CT mice.

Additionally, expression of βGalT1 was increased in the mucosa of CT mice in response to TNF, confirming previous *in vitro* data that TNF induces βGalT1 [10], [11]. Elevated βGalT1 expression might play a role in increased rolling of leukocytes. However, βGalT1-overexpressing mice showed no alterations in rolling of leukocytes. This observation challenges the notion that the role of βGalT1 in inflammation is limited to its effect on the rolling of leukocytes [7], [8]. Apparently, βGalT1 overexpression provides protection through other mechanisms. Modified N-glycosylation caused by TNF-induced βGalT1 expression might provide protection by changing the niche occupied by the bacteria and thereby reducing the chances of systemic bacterial infiltration and the risk of systemic inflammation but more studies are needed to unravel the mechanism in detail. It was recently reported that cytokines such as IL-6 and IL-8 mediate changes in mucin glycosylation. This could be a mechanism that modifies the pattern of glycosylation to increase the anti-inflammatory status of the mucus layer during infection [67], [68].

Mucosal O-glycans are known to be protective in colitis. Mice lacking core-3- and core-2-derived O-glycans are more susceptible to colitis [22], [23]. Mice deficient in core-1-derived O-glycans even develop colitis spontaneously [1]. The N-glycosylation status of the gut has not been studied extensively in the context of colitis. Only recently, it has been shown that the induction of colitis by DSS is slightly reduced in βGalT1 heterozygous mice [24]. However, it must be noted that βGalT1 knockout mice have serious developmental problems and half of them die before weaning. In the surviving mice, proliferation of IEC is augmented and their differentiation is abnormal. Also, these mice have an increased epidermal thickness, which also could be the case in the βGalT1^+/−^ mice [7], and which could explain their partial protection against DSS.

In our studies we show that βGalT1 TG mice are resistant to DSS-induced colitis, which points to the importance of exploring disregulation of βGalT1 expression in colitis patients. Additionally, βGalT1 gene or promoter SNP analysis could reveal if genetic alterations in the major N-galactosylation machinery can be involved in the pathogenesis of colitis. Our data also suggest that therapeutic strategies that enhance the galactosylation state of the gut (such as the use of probiotics or small molecules) could have beneficial effects on disease outcome.

It has been shown that the size of the non-galactosylated N-glycan fraction in human IgG is significantly higher in patients with Crohn's disease. Moreover, βGalT1 expression and activity in B cells are lower in patients with Crohn's disease [69]. We initially hypothesized that higher βGalT1 levels would reduce the amount of non-galactosylated IgGs and thereby protect the TG mice against DSS-induced colitis. However, during the short period of DSS-induced colitis (5 days) no non-galactosylated IgGs were found in CT or TG mice, demonstrating that in our model the galactosylation status of the IgGs cannot be responsible for the observed protection. As a high Firmicutes to Bacteroidetes ratio is believed to be protective in colitis [35], we hypothesize that the protective mechanism resides in the more protective constitution of the microbiota in the gut and further studies (including mice with BGalT overexpression only in the mucosa) are needed to unravel the mechanism in detail.

In summary, βGalT1-overexpressing mice are resistant to TNF-induced SIRS and DSS-induced colitis. We hypothesize that the underlying mechanism is an increase in the galactosylation status of the gut protecting mucus, leading to a healthier Firmicutes/Bacteroidetes ratio in the gut, which protects against inflammatory and chemical insults such as TNF and DSS at the levels of the gut.

## Materials and Methods

### Generation of βGalT1 overexpression mouse

The gateway compatible ROSA26 locus targeting vector for conditionally overexpression of β4GalT1 was generated as previously described (Nyabi et al., 2009). G4 ES cells [70] were grown and manipulated at 37°C in 5% CO2 on mitomycin C-treated mouse embryonic fibroblasts, derived from TgN (DR4)1 Jae embryos, in high glucose DMEM (Invitrogen), supplemented with 15% ES cell-grade FBS (HyClone), 0.1 mM 2-mercaptophenol, 2 mM L-glutamine, 1 mM sodium pyruvate, 0.1 mM non-essential amino acids and 1000 U/ml recombinant LIF (DMBR/VIB Protein Service facility). Parental ES cells were electroporated with the ROSA26 locus targeting vector. G418 resistant ES-cell clones were screened by PCR using both an external (5′ TAGGTAGGGGATCGGGACTCT 3′) and an internal (5′ GGGCATCGACTTCAAGGAGGACGG 3′) primer to generate a 1.5-kb fragment. PCR-positive clones were confirmed by Southern blotting for correct 5′ and 3′ integration and single-copy insertion as described previously (Nyabi et al., 2009). Chimaeras were generated by aggregation of correctly ROSA26-targeted ES cells with Swiss eight-cell stage embryos. Chimeras were crossed with C57BL/6J mice to test for germline transmission.

### Southern blot analysis ES cells

Genomic DNA (10 ug) was digested overnight with 50 Units BamHI in a volume of 45 µl. Samples were loaded on a 0,8% buffer in TAE electrophoresis buffer (0,04 M Tris-acetate, 0,002 M EDTA, pH 8,5) and run at 35 V overnight. The gels were then soaked for 15 min in 0,25 M HCl (depurination buffer). After rinsing with deionised water the gels were denatured for 40 min in 1,5 M NaCl, 0,5 M NaOH, neutralized for 30 min in 1,5 M NaCl, 0,5 M Tris pH 7,2, 0,001 M EDTA pH 8, washed with 2× SSC and blotted overnight with 10× SSC on Hybond N+ membranes (GE Healthcare). The membranes were then washed with 2× SSC, UV cross linked and stored at −20°C. For hybridisation, the membranes were preincubated in Rapid-hyb hybridisation buffer (GE Healthcare) for 1 h at 65°C under rotation. A 5′ external restriction fragment of 550 bp used as hybridisation probe was denatured and labeled with 32P marked dCTP (Perkin Elmer) using the Rediprime II Random Prime Labelling System (GE Healthcare). Labeled probe was purified on MicroSpin S-300 HR colmuns (GE Healthcare), heat denatured, added to the hybridisation buffer and membranes were rotated overnight at 65°C. Membranes were washed with prewarmed washing buffer 0,4×SSC/1%SDS for 15 min and with 0,2×SSC/1%SDS for an additional 15 min at 65°C under shaking. Next, the blots were exposed to a Phosphorimager screen for 3 days. The Phoshor screen was scanned using a Phosphor Imager of Bio-Rad. 5′ integration was confirmed as a 5.8 kb wt allele and 3.0 kb targeted allele.

### PCR for stopcassette and Cre

PCR reaction was performed with Taq Polymerase Kit of Invitrogen (final concentration 1,875 mM MgCl2, 0,5 µM for and rev primer, 0,25 mM XTPs and 0,5 Units Taq polymerase). PCR cycling for stopcassette consisted of denaturation at 94°C for 5 min, 35 cycles of 94°C for 1 min, annealing at 57°C for 1 min and extension at 72°C for 2 min, followed by 10 min at 72°C. PCR cycling for Cre consisted of denaturation at 94°C for 2 min, 29 cycles of 94°C for 30 sec, annealing at 58°C for 30 sec and extension at 72°C for 30 sec, followed by 2 min at 72°C. Primer sequences were used as follows: PCR stopcassette: forward primer GTGATCTGCAACTCCAGTCTTTC; reverse primer CCATCTGCACGAGACTAGTG; PCR Cre: forward primer CGGTCGATGCAACGAGTGATGAGG; reverse primer CCAGAGACGGAAATCCATCGCTCG.

### UDP-Galactosyltransferase Assay in liver

The activity of βGalT1 in the liver was measured in CT versus TG mice. The UDP-galactosyltransferase Assay was performed with the kit of SIGMA (Missouri, USA). The assay is based on the transfer of radioactively labeled galactose from UDP-[galactose-14C] to N-acetyl-D-glucosamine by UDP-galactosyltransferase. The test reaction (with acceptor) consisted of 5 µl of assay buffer, 5 µl of manganese chloride Solution, 5 µl of UDP-Gal Solution (10 mM), 5 µl Acceptor solution, 5 µl radiolabeled UDP-Gal (0,5 uCi) (Perkin Elmer) and 25 µl of protein sample (50 µg) (freshly prepared). A background control (without acceptor) was performed in parallel.

The reactions were incubated at 37°C for 1 hour.

The reaction product, a radiolabeled N-acetyl-D-lactosamine is then purified by ion-exchange chromatography. The eluate is quantified by liquid scintillation counting with a Packard instrument (together with the determination of the total cpm of the radioactive substrate UDP-Gal in a single assay). The βGalT1 activity was 2.5 times increased in the liver of TG compared to CT mice.

### UDP-Galactosyltransferase Assay in ileum and colon

Proteins from the ileum and colon samples of CT and TG mice were isolated with the ProteoExtract Native Membrane Protein Extraction Kit (Calbiochem). Protein concentration was measured with the BCA Protein Assay Kit (Pierce). To measure the galactosyltransferase activity of the protein samples, we used the Glycosyltransferase Activity Kit of R&D (UK).This kit takes advantage of a specific phosphatase to remove inorganic phosphate quantitatively form the leaving nucleotide diphoshate such as GDP, of glycosyltransferase reactions. The released inorganic phosphate is then detected by Malachite Green phosphate detecting reagents. The amount of inorganic phosphate released by the coupling phosphatase is equal to the nucleotide sugar consumed or glycoconjugate product generated; therefore, the rate of inorganic phosphate production reflects the kinetics of the glycosyltransferase reaction.

The reaction consisted of 10 ul UDP-Galactose donor (Calbiochem) (0,5 mM), 10 ul N-Acetyl-D-glucosamine acceptor (Sigma) (5 mM) and 5 ul Coupling Phosphatase 1 (20 ng/ul) in a total volume of 25 ul per well, with addition of 25 ul of the protein sample (4 ug). All samples were assayed in duplicate.

A 2-fold serial dilution was made of a positive control, Recombinant Human β-1,4-Galactosyltransferase (R&D systems) and reactions were initiated with the same donor-acceptor-CP mix. For a negative control, 1× Assay Buffer was used in place of the galactosyltransferase. A 2-fold serial dilution was made of the phosphate standard to generate a standard curve. The reactions were incubated at 37°C for 20 min. Malachite Green reagents were added and the plate was incubated for 20 min at room temperature to stabilize the color development. The optical density of each well was determined using a microplate reader set at 620 nm and the OD was adjusted by subtracting the reading of the negative control.

The corrected ODs of of the positive control samples were plotted against the amounts of galactosyltransferase. The specific activity of the enzyme was calculated using the following equation
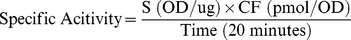
S = Slope of the line

CF = Conversion factor = slope of the linear regression line of the phosphate standard curve

The activity (pmol/min) of galactosyltransferase in the samples was calculated using the regression line of the positive control and this equation.

The βGalT1 activity was 1,5 to 2 times increased in the ileum and colon of TG compared to CT mice.

### Mice

All CT and TG mice were kept in an air-conditioned, temperature-controlled, specific pathogen free animal house under a 12-h light cycle. All mice were born and raised in the same room and had free access to food and water. All experiments were approved by the ethical committee of the Faculty of Sciences of Ghent University.

### Isolation of mucus from the intestine

CT and TG mice were anesthetized and the small intestines and colons were dissected out. The intestines were flushed with ice-cold PBS. Next, the ilea or colons were closed with thread at one end and 1 ml of 10 g/L docusate sodium salt solution was instilled into it. The other end of the intestine was then closed with thread, creating a “sausage”. The intestines were incubated in PBS overnight at RT. The next day, one end of the intestine was cut and the mucus was collected.

### Mucus quantification

Five cm ileal segment was collected, weighted and used for mucus quantification. The ileal section was opened, everted and soaked for 2 h in a 0.1% solution of Alcian blue dissolved in 0.16M sucrose and buffered with 0.05M sodium actetate at a pH of 5.8. Sections were washed in a 0.25 M sucrose solution for 15 minutes and washed in a fresh 0.25 M sucrose solution for 45 minutes to extract uncomplexed dye. Sections were placed in a 10 g/L docusate sodium salt solution overnight at room temperature to extract absorbed dye from the tissue and then centrifuged at 700×g. 100 ul of each sample (in triplicate) was plated on a 96-well plate and the density was measured at 620 nm to determine the amount of absorbed dye in each sample. The results are reported as micrograms of alcian blue/gram of ileum.

### N-glycosylation profiling of mucus

The mucus protein concentration was measured and 120 µg was used for isolation of N-glycans, which were separated according to size by DSA-FACE as described previously [32]. To verify the peaks contain structures with terminal GlcNAc rather than galactose, we performed glycodigests with Streptococcus pneumonia b-1,4-galactosidase (2 mU/digest) and jack bean b-N-acetylhexosaminidase (10 mU/digest) (all from Prozyme, San Leandro, CA, USA).

### Isolation of mucosa of mice

CT and TG mice were anesthetized and ileum was removed and flushed with ice-cold PBS. It was cut along its length and the mucosa was scraped off with a microscope slide. On histological examination of the intestine it was noted that this technique removed mainly the mucosa, leaving most of the submucosa and all of the deeper structures intact.

### RNA isolation, cDNA synthesis and qPCR on mucosal samples

RNA of mucosa was prepared with the RNeasy Mini kit (Qiagen Benelux B.V.,Venlo, the Netherlands). DNase digestion was performed with the RNase-free DNase Set of Qiagen. cDNA was prepared with the iScript cDNA synthesis kit of Bio-Rad starting from 2 µg of total RNA. Real-time qPCR using the Light Cycler 480 (Roche Diagnostics) was performed with a fivefold dilution of the cDNA. Each 10 µL-assay contained 5 µl of 2× Fast SYBR Green Master Mix (Applied Biosystems, Nieuwerkerk Ad Ijssel, The Netherlands), 2 µL primer mix for each gene (0.5 µM each final concentration), and 3 µL of diluted cDNA. The primers were designed with the PrimerExpress software or selected from the Primerbank of Harvard. A geometric averaging method (GeNorm) identified the best performing housekeeping genes (UBC and GAPDH). Each reaction was performed in triplicate. PCR cycling consisted of denaturation at 95°C for 5 min, 50 cycles of 95°C for 10 s and 60°C for 30 s, and detection for 1 s at 72°C. Results are given as relative expression values normalized to the geometric mean of the housekeeping genes. Primers sequences used were as follows: mouse βGalT1-forw: AAAATTCGCTGTGGCCCTC, mouse βGalT1-rev: GGCACAATCATGCTGGAGAA.

### In vivo TNF toxicity

Recombinant mouse TNF was produced in *E. coli* and purified to homogeneity in our laboratory. It had a specific activity of 9.46×10^7^ IU/mg and endotoxin levels did not exceed 1 ng/ml of protein. Mice were injected i.p. with a LD_50_ of TNF. Body temperature and survival were monitored and blood for cytokine analysis was collected.

### Interleukin-6 measurement

To measure interleukin-6 (IL-6) in serum, we used the 7TD1 hybridoma cell line, which proliferates in response to IL-6 [71]. In the presence of serially diluted serum or mouse IL-6 as a standard, 7000 cells per well were cultured in 96-well plates. After 72 h of culture, the number of cells was determined by a hexosaminidase colorimetric method. The assay has a detection limit of 1 pg/ml.

### Hematoxylin-eosin, CD45 staining, Ki67 staining, PAS staining and MMP-7 staining

Mice were anesthetized with ketamine xylazine. The ilea or colons were removed and flushed with cold PBS, after which they were fixed in 4% paraformaldehyde for 24 h at 4°C. Sections of 5 µm were cut from ilea embedded in paraffin and stained with hematoxylin (Fluka) and eosin (Merck).

For PAS staining, ileum sections were deparaffinized and stained with the periodic acid-schiff kit (Sigma - Aldrich N.V., 395-B).

For immunolabeling of CD45 and Ki67, ileum sections were deparaffinized and then boiled in citrate buffer. Sections were rinsed and incubated in 0.3% H_2_O_2_ to quench endogenous peroxidases. Next, sections were incubated in blocking buffer (10 mM Tris-HCl pH 7.4; 0.1 M MgCl_2_; 0.5% Tween20; 1% BSA; 5% serum) for 1 h at room temperature, followed by overnight incubation at 4°C with anti-CD45 (1∶250; 550539, Pharmingen) or anti-Ki67 primary antibody (1/30; 7249, DAKO). After rinsing with PBS/0.1% Tween20 (PBS-T), sections were incubated with biotinylated secondary antibody (1/300; DAKO) for 30 min at room temperature. They were again rinsed in PBS-T and incubated with avidin/biotin complex (Vectastain Elite ABC kit, Vector Labs) for 20 min. After rinsing in PBS-T, sections were incubated in AEC (Dako) for 15 min. Finally, they were counterstained with Gills III and mounted with Aquatex.

For MMP-7 immunostaining, ileum sections were dewaxed and boiled in 10 mM sodium citrate buffer for antigen retrieval, incubated for 1 h in blocking buffer (10 mM Tris-HCl pH 7.4, 0.1 M MgCl2, 0.5% Tween-20, 1% BSA and 5% serum) and incubated with MMP7 (1/100; 3801; Cell Signalling Technology) antibody.

Fluorescent images and light microscopy images were taken by a laser scanning confocal microscope (Leica TCS SP5) and an Olympus light microscope respectively.

### Fecal DNA extraction

Fresh feces were obtained from female cohoused CT and TG mice of 12 weeks old and stored immediately at −80°C. DNA was extracted from the feces using the QIAamp DNA Stool Mini Kit (Qiagen, Venlo, The Netherlands).

### Real-time qPCR analysis on fecal DNA

qPCR amplification and detection were performed using the Roche LightCycler 480 and its SYBR Green I Master kit. Each PCR reaction included 300 nM of forward and reverse primers, 9 ng of DNA, and SYBR Green Master Mix. Samples without template served as negative controls. Samples were incubated at 95°C for 5 min and then amplified through 45 cycles of 95°C for 10 s, 60°C for 30 s, and 72°C for 1 s. The amount of Firmicutes and Bacteroidetes 16S rRNA in each sample was normalized to the total amount of 16S rRNA amplified with universal primers (the total amount of bacteria was not different between CT and TG mice as demonstrated by the QPCR with universal 16S rRNA). Primers used in these experiments are the following: Bacteroidetes: BactF GGARCATGTGGTTTAATTCGATGAT; BactR AGCTGACGACAACCATGCAG; Firmicutes: FirmF GGAGYATGTGGTTTAATTCGAAGCA; FirmR AGCTGACGACAACCATGCAC; Total Bacteria: EubF ACTCCTACGGGAGGCAGCAG; EubR ATTACCGCGGCTGCTGG.

### Induction of DSS-induced colitis and clinical score

Female and male CT and TG mice at the age of 9–11 weeks were used in DSS-induced colitis experiments. Acute colitis was induced by addition of 3% DSS (36–50 kD; MP Biomedicals) to the drinking water for five days. Body weight, occult or gross blood loss from the rectum, and stool consistency were determined daily. Fecal blood was determined using Hemoccult SENSA (Beckman Coulter). The baseline clinical score was determined on day 0. In brief, no weight loss was scored as 0, weight loss of 1–5% of baseline as 1, 5–10% as 2, 10–20% as 3, and >20% as 4. For bleeding, a score of 0 was assigned for no blood, 2 for positive hemoccult, and 4 for gross bleeding. For stool consistency, a score of 0 was assigned for well-formed pellets, 2 for pasty and semiformed stools, and 4 for liquid stools. For each mouse, these scores were combined and divided by three to give an overall clinical score ranging from 0 (healthy) to 4 (maximal colitis). To eliminate observer bias, scoring was blinded. Postmortem, the entire colon was removed from cecum to anus and its length was measured as an indicator of inflammation.

### Gut permeability test

FITC-labeled dextran (4 kDa, Sigma) was administered to mice by gavage at 150 mg/kg body weight. Five hours later, blood collected by heart puncture in EDTA-coated tubes (Sarstedt) and plasma was prepared. Leakage of FITC-labeled dextran into the circulation was determined by measurement of fluorescence with λ_ex_ = 488 nm and λ_em_ = 520 nm. Values were normalized to the lowest value.

### Statistics

Data were expressed as means ± standard errors of the means (SEM). Statistical significance between two groups was evaluated with a two-tailed t-test with a 95% confidence interval (CI). The log rank test was used for analysis of survival curves. All tests were calculated with Prism software. p values<0.05 were considered statistically significant. P values are indicated as *, **, and *** to represent 0.01≤p<0.05, 0.001≤p<0.01, and p<0.001, respectively.

## Supporting Information

Figure S1 Relative βGalT1 gene expression and activity in CT versus TG mice. (A) Relative βGalT1 gene expression in the liver of TG mice (n = 4) is 2 fold higher compared to CT mice (n = 4). (B) Relative βGalT1 gene expression in the brain of TG mice (n = 4) is 2 fold higher compared to CT mice (n = 4). (C) βGalT1 activity in the liver of TG mice (n = 4) is upregulated 2 fold compared to CT mice (n = 4).
(TIF)

Figure S2 Blood composition of CT versus TG mice measured by Hemavet. Twenty µl of fresh blood from unstimulated CT and TG mice, was obtained in EDTA-coated vials and immediately used for measuring blood composition by Hemavet (Scientific Inc. Oxford).
(TIF)

Figure S3 Mucus in intestine of CT versus TG mice. Staining of the small intestine (without stimulus) with Alcian blue to visualize the goblet cells in CT (A left) and TG (A right) mice. The quantification was done using the program Velocity. The bar in the left corner represents 50 µm (B) Amount of mucus present in the intestine of CT versus TG mice. No differences were observed between CT and TG mice. (C) Analysis of the N-glycosylation profile of colon mucus from mice with and without hexosaminidase treatment. Top panel is the N-glycosylation profile of a standard protein, RNase B. The second panel represents a representative sample of colon mucus of a CT mouse. The third panel represents the N-glycosylation profile of CT mucus (the same sample as shown in panel 2), but after hexosaminidase treatment. The fourth panel is a representative sample of colon mucus of a TG mouse. The fifth panel is the same sample as in the fourth panel but after hexosaminidase treatment, demonstrating that TG mucus has less structures ending with GlcNac compared to CT mucus. (D) Analysis of the N-glycosylation profile of colon mucus from mice with and without ß-galactosidase treatment. After isolation of the N-glycans out of colon mucus, these N-glycans are treated with ß-galactosidase in order to identify terminal galoctose residues in the sample since ß-galactosidase only cleaves terminal galactose. Top panel is the N-glycosylation profile of RNase B. The second panel represents a typical sample of colon mucus of a CT mouse. The third panel is the same sample as the second panel but treated with ß-galactosidase. Only a few peaks shift, which demonstrates that only a small amount of the N-glycans in CT mucus have terminal galactose. The fourth panel is a representative sample of colon mucus of a TG mouse. The fifth panel is the same sample as in the fourth panel but treated with ß-galactosidase. Here a clear shift of peaks is observed demonstrating that the N-glycans in TG colon mucus have more terminal galactose compared to the CT colon mucus.
(TIF)

Table S1 Primers used for the qPCR measurement of bacteria in the gut of mice.
(TIF)

## References

[1] Fu J, Wei B, Wen T, Johansson ME, Liu X, et al (2011) Loss of intestinal core 1-derived O-glycans causes spontaneous colitis in mice. The Journal of clinical investigation 121: 1657–1666. 21383503 10.1172/JCI45538 PMC3069788

[2] McGuckin MA, Linden SK, Sutton P, Florin TH (2011) Mucin dynamics and enteric pathogens. Nat Rev Microbiol 9: 265–278. 21407243 10.1038/nrmicro2538

[3] Thornton DJ, Rousseau K, McGuckin MA (2008) Structure and function of the polymeric mucins in airways mucus. Annu Rev Physiol 70: 459–486. 17850213 10.1146/annurev.physiol.70.113006.100702

[4] Macao B, Johansson DG, Hansson GC, Hard T (2006) Autoproteolysis coupled to protein folding in the SEA domain of the membrane-bound MUC1 mucin. Nat Struct Mol Biol 13: 71–76. 16369486 10.1038/nsmb1035

[5] Kotani N, Asano M, Iwakura Y, Takasaki S (2001) Knockout of mouse beta 1,4-galactosyltransferase-1 gene results in a dramatic shift of outer chain moieties of N-glycans from type 2 to type 1 chains in hepatic membrane and plasma glycoproteins. The Biochemical journal 357: 827–834. 11463354 10.1042/0264-6021:3570827 PMC1222013

[6] Hennet T (2002) The galactosyltransferase family. Cell Mol Life Sci 59: 1081–1095. 12222957 10.1007/s00018-002-8489-4 PMC11337546

[7] Asano M, Furukawa K, Kido M, Matsumoto S, Umesaki Y, et al (1997) Growth retardation and early death of beta-1,4-galactosyltransferase knockout mice with augmented proliferation and abnormal differentiation of epithelial cells. EMBO J 16: 1850–1857. 9155011 10.1093/emboj/16.8.1850 PMC1169788

[8] Asano M, Nakae S, Kotani N, Shirafuji N, Nambu A, et al (2003) Impaired selectin-ligand biosynthesis and reduced inflammatory responses in beta-1,4-galactosyltransferase-I-deficient mice. Blood 102: 1678–1685. 12714507 10.1182/blood-2003-03-0836

[9] Van Hauwermeiren F, Vandenbroucke RE, Libert C (2011) Treatment of TNF mediated diseases by selective inhibition of soluble TNF or TNFR1. Cytokine Growth Factor Rev 22: 311–319. 21962830 10.1016/j.cytogfr.2011.09.004

[10] Xu D, Cui Z, Liu W, Tao R, Tao T, et al (2011) Tumor necrosis factor-alpha up-regulates the expression of beta1,4-galactosyltransferase-I in human fibroblast-like synoviocytes. Inflammation 34: 531–538. 20886274 10.1007/s10753-010-9260-x

[11] Yan M, Xia C, Niu S, Shao X, Cheng C, et al (2007) The role of TNF-alpha and its receptors in the production of beta-1,4 galactosyltransferase I and V mRNAs by rat primary astrocytes. J Mol Neurosci 33: 155–162. 17917074 10.1007/s12031-007-0033-4

[12] Yuan Q, Yang H, Cheng C, Li C, Wu X, et al (2012) beta-1,4-Galactosyltransferase I involved in Schwann cells proliferation and apoptosis induced by tumor necrosis factor-alpha via the activation of MAP kinases signal pathways. Mol Cell Biochem 365: 149–158. 22359038 10.1007/s11010-012-1254-6

[13] Niu S, Fei M, Cheng C, Yan M, Gao S, et al (2008) Altered beta-1,4-galactosyltransferase I expression during early inflammation after spinal cord contusion injury. J Chem Neuroanat 35: 245–256. 18294815 10.1016/j.jchemneu.2008.01.002

[14] Shen A, Chen J, Qian J, Zhu J, Hu L, et al (2009) Elevated beta1,4-galactosyltransferase-I induced by the intraspinal injection of lipopolysaccharide. Glycoconj J 26: 19–31. 18677561 10.1007/s10719-008-9158-0

[15] Dharmani P, Leung P, Chadee K (2011) Tumor necrosis factor-alpha and Muc2 mucin play major roles in disease onset and progression in dextran sodium sulphate-induced colitis. PLoS One 6: e25058. 21949848 10.1371/journal.pone.0025058 PMC3176316

[16] Elson CO, Cong Y, McCracken VJ, Dimmitt RA, Lorenz RG, et al (2005) Experimental models of inflammatory bowel disease reveal innate, adaptive, and regulatory mechanisms of host dialogue with the microbiota. Immunol Rev 206: 260–276. 16048554 10.1111/j.0105-2896.2005.00291.x

[17] Van Hauwermeiren FAM, Karagianni N, Kranidioti K, Vandenbroucke RE, Loges S, et al (2013) Safe TNF-based antitumor therapy following p55TNFR reduction in intestinal epithelium. J Clin Invest May 15. pii: 65624. doi: 10.1172/JCI65624. [Epub ahead of print] 10.1172/JCI65624 PMC3668821 23676465

[18] Podolsky DK, Isselbacher KJ (1984) Glycoprotein composition of colonic mucosa. Specific alterations in ulcerative colitis. Gastroenterology 87: 991–998. 6090262

[19] Rhodes JM (1997) Colonic mucus and ulcerative colitis. Gut 40: 807–808. 9245940 10.1136/gut.40.6.807 PMC1027212

[20] Heazlewood CK, Cook MC, Eri R, Price GR, Tauro SB, et al (2008) Aberrant mucin assembly in mice causes endoplasmic reticulum stress and spontaneous inflammation resembling ulcerative colitis. PLoS Med 5: e54. 18318598 10.1371/journal.pmed.0050054 PMC2270292

[21] Van der Sluis M, De Koning BA, De Bruijn AC, Velcich A, Meijerink JP, et al (2006) Muc2-deficient mice spontaneously develop colitis, indicating that MUC2 is critical for colonic protection. Gastroenterology 131: 117–129. 16831596 10.1053/j.gastro.2006.04.020

[22] An G, Wei B, Xia B, McDaniel JM, Ju T, et al (2007) Increased susceptibility to colitis and colorectal tumors in mice lacking core 3-derived O-glycans. The Journal of experimental medicine 204: 1417–1429. 17517967 10.1084/jem.20061929 PMC2118614

[23] Stone EL, Ismail MN, Lee SH, Luu Y, Ramirez K, et al (2009) Glycosyltransferase function in core 2-type protein O glycosylation. Mol Cell Biol 29: 3770–3782. 19349303 10.1128/MCB.00204-09 PMC2698761

[24] Shinzaki S, Iijima H, Fujii H, Kuroki E, Tatsunaka N, et al (2012) Altered Oligosaccharide Structures Reduce Colitis Induction in Mice Defective in beta-1,4-Galactosyltransferase. Gastroenterology 10.1053/j.gastro.2012.02.008 22333949

[25] Nyabi O, Naessens M, Haigh K, Gembarska A, Goossens S, et al (2009) Efficient mouse transgenesis using Gateway-compatible ROSA26 locus targeting vectors and F1 hybrid ES cells. Nucleic Acids Res 37: e55. 19279185 10.1093/nar/gkp112 PMC2673446

[26] Betz UA, Vosshenrich CA, Rajewsky K, Muller W (1996) Bypass of lethality with mosaic mice generated by Cre-loxP-mediated recombination. Curr Biol 6: 1307–1316. 8939573 10.1016/s0960-9822(02)70717-3

[27] Linden SK, Sutton P, Karlsson NG, Korolik V, McGuckin MA (2008) Mucins in the mucosal barrier to infection. Mucosal Immunol 1: 183–197. 19079178 10.1038/mi.2008.5 PMC7100821

[28] Callewaert N, Geysens S, Molemans F, Contreras R (2001) Ultrasensitive profiling and sequencing of N-linked oligosaccharides using standard DNA-sequencing equipment. Glycobiology 11: 275–281. 11358876 10.1093/glycob/11.4.275

[29] Laroy W, Contreras R, Callewaert N (2006) Glycome mapping on DNA sequencing equipment. Nat Protoc 1: 397–405. 17406262 10.1038/nprot.2006.60

[30] Vanhooren V, Dewaele S, Kuro OM, Taniguchi N, Dolle L, et al (2011) Alteration in N-glycomics during mouse aging: a role for FUT8. Aging Cell 10: 1056–1066. 21951615 10.1111/j.1474-9726.2011.00749.x

[31] Vanhooren V, Dewaele S, Libert C, Engelborghs S, De Deyn PP, et al (2010) Serum N-glycan profile shift during human ageing. Exp Gerontol 45: 738–743. 20801208 10.1016/j.exger.2010.08.009

[32] Vanhooren V, Laroy W, Libert C, Chen C (2008) N-glycan profiling in the study of human aging. Biogerontology 9: 351–356. 18431686 10.1007/s10522-008-9140-z

[33] Vanhooren V, Liu XE, Franceschi C, Gao CF, Libert C, et al (2009) N-glycan profiles as tools in diagnosis of hepatocellular carcinoma and prediction of healthy human ageing. Mech Ageing Dev 130: 92–97. 19070631 10.1016/j.mad.2008.11.008

[34] Moran AP, Gupta A, Joshi L (2011) Sweet-talk: role of host glycosylation in bacterial pathogenesis of the gastrointestinal tract. Gut 60: 1412–1425. 21228430 10.1136/gut.2010.212704

[35] Sokol H, Seksik P, Furet JP, Firmesse O, Nion-Larmurier I, et al (2009) Low counts of Faecalibacterium prausnitzii in colitis microbiota. Inflamm Bowel Dis 15: 1183–1189. 19235886 10.1002/ibd.20903

[36] Mariat D, Firmesse O, Levenez F, Guimaraes V, Sokol H, et al (2009) The Firmicutes/Bacteroidetes ratio of the human microbiota changes with age. BMC Microbiol 9: 123. 19508720 10.1186/1471-2180-9-123 PMC2702274

[37] Ayabe T, Satchell DP, Wilson CL, Parks WC, Selsted ME, et al (2000) Secretion of microbicidal alpha-defensins by intestinal Paneth cells in response to bacteria. Nat Immunol 1: 113–118. 11248802 10.1038/77783

[38] Ayabe T, Satchell DP, Pesendorfer P, Tanabe H, Wilson CL, et al (2002) Activation of Paneth cell alpha-defensins in mouse small intestine. J Biol Chem 277: 5219–5228. 11733520 10.1074/jbc.M109410200

[39] Vandenbroucke RE VI, Van Hauwermeiren F, Van Wonterghem E, Wilson C, Libert C (2013) Pro-inflammatory effects of matrix metalloproteinase 7 in acute inflammation. Mucosal Immunology In press. 10.1038/mi.2013.76 24129163

[40] Tracey KJ, Beutler B, Lowry SF, Merryweather J, Wolpe S, et al (1986) Shock and tissue injury induced by recombinant human cachectin. Science 234: 470–474. 3764421 10.1126/science.3764421

[41] Ma TY, Boivin MA, Ye D, Pedram A, Said HM (2005) Mechanism of TNF-{alpha} modulation of Caco-2 intestinal epithelial tight junction barrier: role of myosin light-chain kinase protein expression. Am J Physiol Gastrointest Liver Physiol 288: G422–430. 15701621 10.1152/ajpgi.00412.2004

[42] Marchiando AM, Shen L, Graham WV, Weber CR, Schwarz BT, et al (2010) Caveolin-1-dependent occludin endocytosis is required for TNF-induced tight junction regulation in vivo. J Cell Biol 189: 111–126. 20351069 10.1083/jcb.200902153 PMC2854371

[43] Vereecke L, Sze M, Mc Guire C, Rogiers B, Chu Y, et al (2010) Enterocyte-specific A20 deficiency sensitizes to tumor necrosis factor-induced toxicity and experimental colitis. The Journal of experimental medicine 207: 1513–1523. 20530205 10.1084/jem.20092474 PMC2901067

[44] Libert C, Brouckaert P, Shaw A, Fiers W (1990) Induction of interleukin 6 by human and murine recombinant interleukin 1 in mice. Eur J Immunol 20: 691–694. 2318254 10.1002/eji.1830200333

[45] Takahashi N, Vanlaere I, de Rycke R, Cauwels A, Joosten LA, et al (2008) IL-17 produced by Paneth cells drives TNF-induced shock. The Journal of experimental medicine 205: 1755–1761. 18663129 10.1084/jem.20080588 PMC2525583

[46] Van Molle W, Wielockx B, Mahieu T, Takada M, Taniguchi T, et al (2002) HSP70 protects against TNF-induced lethal inflammatory shock. Immunity 16: 685–695. 12049720 10.1016/s1074-7613(02)00310-2

[47] Sokol H, Pigneur B, Watterlot L, Lakhdari O, Bermudez-Humaran LG, et al (2008) Faecalibacterium prausnitzii is an anti-inflammatory commensal bacterium identified by gut microbiota analysis of Crohn disease patients. Proc Natl Acad Sci U S A 105: 16731–16736. 18936492 10.1073/pnas.0804812105 PMC2575488

[48] Dieleman LA, Ridwan BU, Tennyson GS, Beagley KW, Bucy RP, et al (1994) Dextran sulfate sodium-induced colitis occurs in severe combined immunodeficient mice. Gastroenterology 107: 1643–1652. 7958674 10.1016/0016-5085(94)90803-6

[49] Okayasu I, Hatakeyama S, Yamada M, Ohkusa T, Inagaki Y, et al (1990) A novel method in the induction of reliable experimental acute and chronic ulcerative colitis in mice. Gastroenterology 98: 694–702. 1688816 10.1016/0016-5085(90)90290-h

[50] Iwaya H, Maeta K, Hara H, Ishizuka S (2012) Mucosal permeability is an intrinsic factor in susceptibility to dextran sulfate sodium-induced colitis in rats. Exp Biol Med (Maywood) 10.1258/ebm.2011.011269 22522346

[51] Vandenbroucke RE, Dejonckheere E, Lodens S, Van Hauwermeiren F, De Rycke R, et al (2013) MMP13 modulates intestinal epithelial barrier integrity in inflammatory diseases. EMBO Mol Med in press. 10.1002/emmm.201202100 PMC3721470 23723167

[52] Kukuruzinska MA, Lennon K (1998) Protein N-glycosylation: molecular genetics and functional significance. Crit Rev Oral Biol Med 9: 415–448. 9825220 10.1177/10454411980090040301

[53] Ohtsubo K, Marth JD (2006) Glycosylation in cellular mechanisms of health and disease. Cell 126: 855–867. 16959566 10.1016/j.cell.2006.08.019

[54] Raman R, Raguram S, Venkataraman G, Paulson JC, Sasisekharan R (2005) Glycomics: an integrated systems approach to structure-function relationships of glycans. Nat Methods 2: 817–824. 16278650 10.1038/nmeth807

[55] Furukawa K, Sato T (1999) Beta-1,4-galactosylation of N-glycans is a complex process. Biochim Biophys Acta 1473: 54–66. 10580129 10.1016/s0304-4165(99)00169-5

[56] Rini J, Esko J, Varki A (2009) Glycosyltransferases and Glycan-processing Enzymes. 20301247

[57] Varki A, Lowe JB (2009) Biological Roles of Glycans. 20301233

[58] Mori R, Kondo T, Nishie T, Ohshima T, Asano M (2004) Impairment of skin wound healing in beta-1,4-galactosyltransferase-deficient mice with reduced leukocyte recruitment. Am J Pathol 164: 1303–1314. 15039218 10.1016/s0002-9440(10)63217-8 PMC1615332

[59] Strugala V, Allen A, Dettmar PW, Pearson JP (2003) Colonic mucin: methods of measuring mucus thickness. Proc Nutr Soc 62: 237–243. 12756973 10.1079/pns2002205

[60] Rozee KR, Cooper D, Lam K, Costerton JW (1982) Microbial flora of the mouse ileum mucous layer and epithelial surface. Appl Environ Microbiol 43: 1451–1463. 7103492 10.1128/aem.43.6.1451-1463.1982 PMC244253

[61] Littman DR, Pamer EG (2011) Role of the commensal microbiota in normal and pathogenic host immune responses. Cell Host Microbe 10: 311–323. 22018232 10.1016/j.chom.2011.10.004 PMC3202012

[62] Sando L, Pearson R, Gray C, Parker P, Hawken R, et al (2009) Bovine Muc1 is a highly polymorphic gene encoding an extensively glycosylated mucin that binds bacteria. J Dairy Sci 92: 5276–5291. 19762846 10.3168/jds.2009-2216

[63] Schroers V, Van Der Marel M, Steinhagen D (2008) Influence of carp intestinal mucus molecular size and glycosylation on bacterial adhesion. Dis Aquat Organ 81: 135–142. 18924378 10.3354/dao01947

[64] Magalhaes A, Gomes J, Ismail MN, Haslam SM, Mendes N, et al (2009) Fut2-null mice display an altered glycosylation profile and impaired BabA-mediated Helicobacter pylori adhesion to gastric mucosa. Glycobiology 19: 1525–1536. 19706747 10.1093/glycob/cwp131 PMC2782244

[65] Sonnenburg JL, Xu J, Leip DD, Chen CH, Westover BP, et al (2005) Glycan foraging in vivo by an intestine-adapted bacterial symbiont. Science 307: 1955–1959. 15790854 10.1126/science.1109051

[66] Keshav S (2006) Paneth cells: leukocyte-like mediators of innate immunity in the intestine. J Leukoc Biol 80: 500–508. 16793911 10.1189/jlb.1005556

[67] Groux-Degroote S, Krzewinski-Recchi MA, Cazet A, Vincent A, Lehoux S, et al (2008) IL-6 and IL-8 increase the expression of glycosyltransferases and sulfotransferases involved in the biosynthesis of sialylated and/or sulfated Lewisx epitopes in the human bronchial mucosa. The Biochemical journal 410: 213–223. 17944600 10.1042/BJ20070958

[68] Wu YM, Nowack DD, Omenn GS, Haab BB (2009) Mucin glycosylation is altered by pro-inflammatory signaling in pancreatic-cancer cells. J Proteome Res 8: 1876–1886. 19714813 10.1021/pr8008379 PMC2893235

[69] Shinzaki S, Iijima H, Nakagawa T, Egawa S, Nakajima S, et al (2008) IgG oligosaccharide alterations are a novel diagnostic marker for disease activity and the clinical course of inflammatory bowel disease. Am J Gastroenterol 103: 1173–1181. 18177457 10.1111/j.1572-0241.2007.01699.x

[70] George SH, Gertsenstein M, Vintersten K, Korets-Smith E, Murphy J, et al (2007) Developmental and adult phenotyping directly from mutant embryonic stem cells. Proc Natl Acad Sci U S A 104: 4455–4460. 17360545 10.1073/pnas.0609277104 PMC1838622

[71] Van Snick J, Cayphas S, Vink A, Uyttenhove C, Coulie PG, et al (1986) Purification and NH2-terminal amino acid sequence of a T-cell-derived lymphokine with growth factor activity for B-cell hybridomas. Proc Natl Acad Sci U S A 83: 9679–9683. 2948184 10.1073/pnas.83.24.9679 PMC387204

