# Supplementary figures and images for: Mice Overexpressing β-1,4-Galactosyltransferase I Are Resistant to TNF-Induced Inflammation and DSS-Induced Colitis
Source: PLoS One. 2013 Dec 5;8(12):e79883. doi: 10.1371/journal.pone.0079883 (PMC3855152; doi:10.1371/journal.pone.0079883)

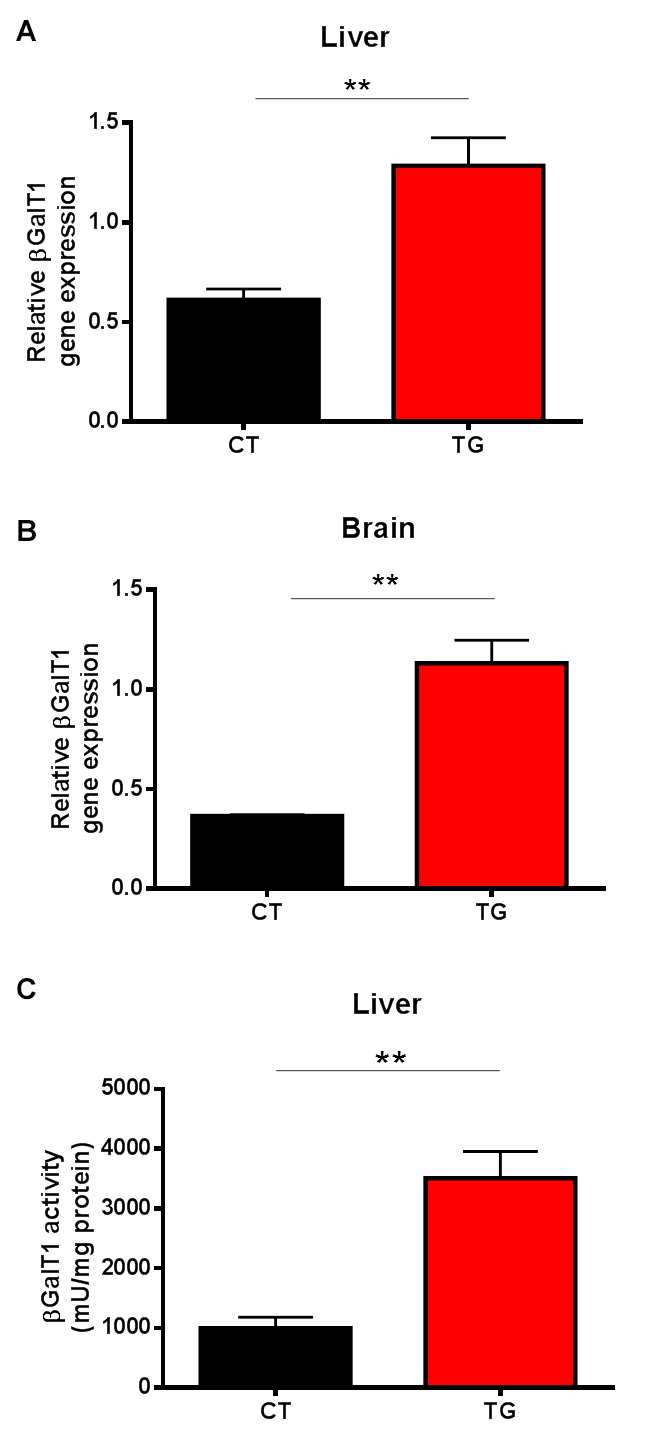

Supplement: Figure S1 — Relative βGalT1 gene expression and activity in CT versus TG mice. (A) Relative βGalT1 gene expression in the liver of TG mice (n = 4) is 2 fold higher compared to CT mice (n = 4). (B) Relative βGalT1 gene expression in the brain of TG mice (n = 4) is 2 fold higher compared to CT mice (n = 4). (C) βGalT1 activity in the liver of TG mice (n = 4) is upregulated 2 fold compared to CT mice (n = 4). (TIF) [file pone.0079883.s001.tif]

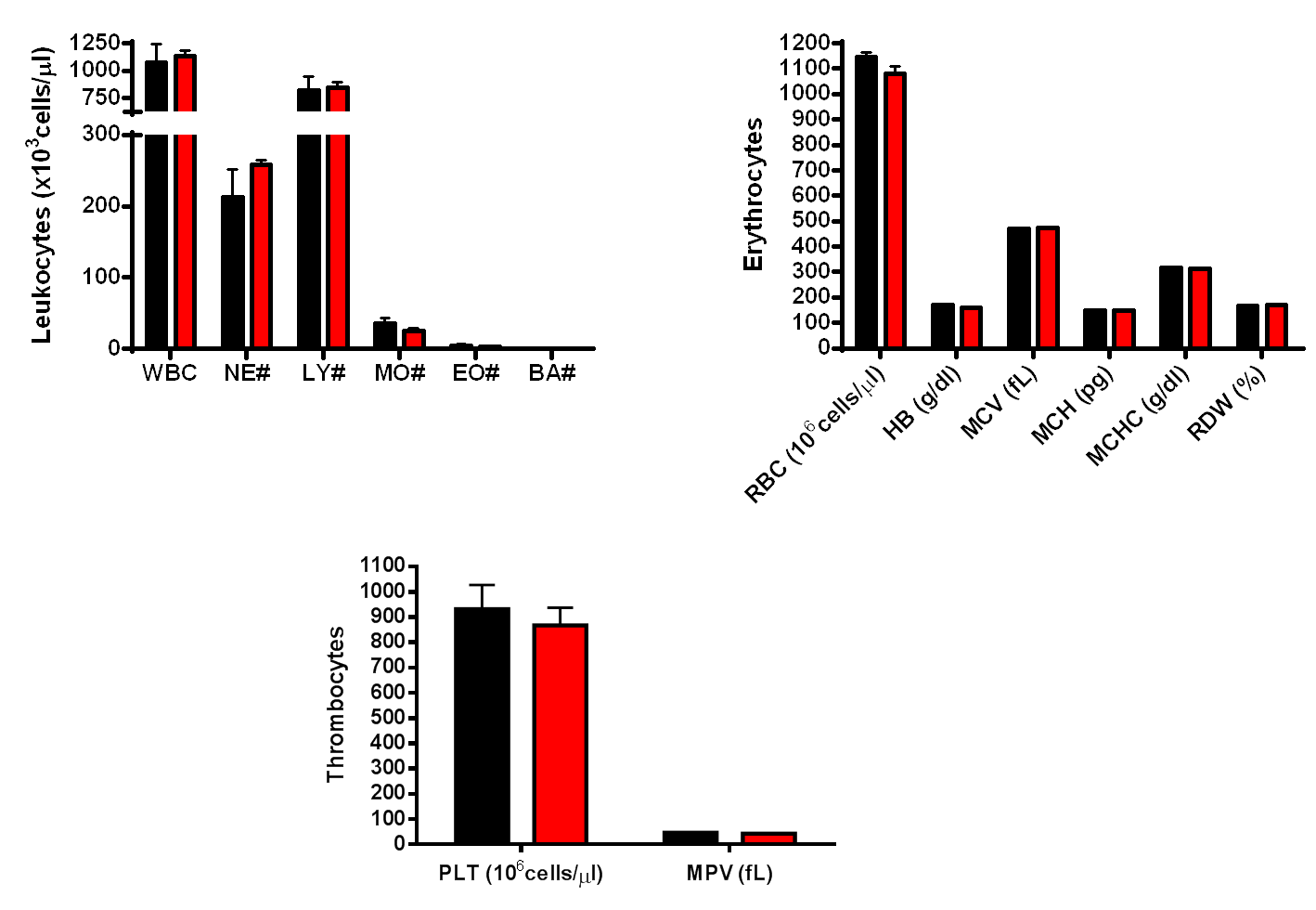

Supplement: Figure S2 — Blood composition of CT versus TG mice measured by Hemavet. Twenty µl of fresh blood from unstimulated CT and TG mice, was obtained in EDTA-coated vials and immediately used for measuring blood composition by Hemavet (Scientific Inc. Oxford). (TIF) [file pone.0079883.s002.tif]

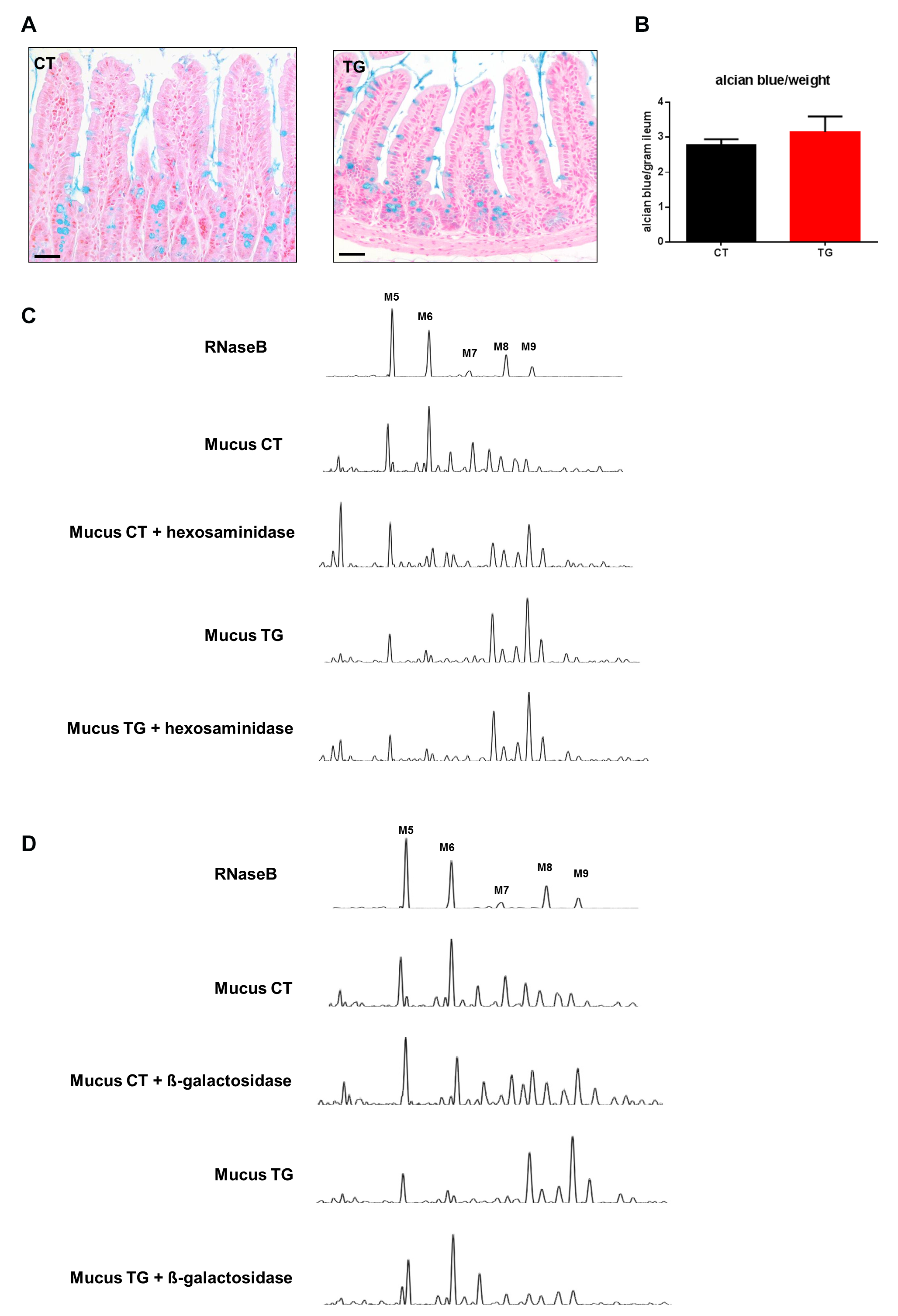

Supplement: Figure S3 — Mucus in intestine of CT versus TG mice. Staining of the small intestine (without stimulus) with Alcian blue to visualize the goblet cells in CT (A left) and TG (A right) mice. The quantification was done using the program Velocity. The bar in the left corner represents 50 µm (B) Amount of mucus present in the intestine of CT versus TG mice. No differences were observed between CT and TG mice. (C) Analysis of the N-glycosylation profile of colon mucus from mice with and without hexosaminidase treatment. Top panel is the N-glycosylation profile of a standard protein, RNase B. The second panel represents a representative sample of colon mucus of a CT mouse. The third panel represents the N-glycosylation profile of CT mucus (the same sample as shown in panel 2), but after hexosaminidase treatment. The fourth panel is a representative sample of colon mucus of a TG mouse. The fifth panel is the same sample as in the fourth panel but after hexosaminidase treatment, demonstrating that TG mucus has less structures ending with GlcNac compared to CT mucus. (D) Analysis of the N-glycosylation profile of colon mucus from mice with and without ß-galactosidase treatment. After isolation of the N-glycans out of colon mucus, these N-glycans are treated with ß-galactosidase in order to identify terminal galoctose residues in the sample since ß-galactosidase only cleaves terminal galactose. Top panel is the N-glycosylation profile of RNase B. The second panel represents a typical sample of colon mucus of a CT mouse. The third panel is the same sample as the second panel but treated with ß-galactosidase. Only a few peaks shift, which demonstrates that only a small amount of the N-glycans in CT mucus have terminal galactose. The fourth panel is a representative sample of colon mucus of a TG mouse. The fifth panel is the same sample as in the fourth panel but treated with ß-galactosidase. Here a clear shift of peaks is observed demonstrating that the N-glycans in TG colon mucus have mor [file pone.0079883.s003.tif]
